# Is the Timing of Surgery a Sufficient Predictive Factor for Outcomes in Patients with Proximal Femur Fractures? A Systematic Review

**DOI:** 10.3390/jpm14070773

**Published:** 2024-07-21

**Authors:** Mihai Rădulescu, Bogdan-Radu Necula, Sandu Aurel Mironescu, Mihai Dan Roman, Alexander Schuh, Radu-Dan Necula

**Affiliations:** 1 Faculty of Medicine, Transilvania University of Brașov, 500036 Brașov, Romania; 2 Faculty of Medicine, Lucian Blaga University of Sibiu, 550169 Sibiu, Romania; 3 Department of Musculoskeletal Research, Marktredwitz Hospital, 95615 Marktredwitz, Germany

**Keywords:** hip fracture, time to surgery, hip arthroplasty, length of stay, delayed surgery

## Abstract

(1) Background: Hip fractures are currently recognized as major public health problems, raising many issues in terms of both patients’ quality of life and the cost associated with caring for this type of fracture. Many authors debate whether to operate as soon as possible or to postpone surgery until the patient is stable. The purpose of this review was to review the literature and obtain additional information about the moment of surgery, the time to surgery, length of hospital stay, and how all of these factors influence patient mortality and complications. (2) Methods: The systematic search was conducted according to the Preferred Reporting Items for Systematic Reviews and Meta-Analyses (PRISMA) and PICO guidelines, using the Google Scholar platform, for articles published between 2015 and 2023. Quality assessment was performed. (3) Results: After applying the inclusion criteria, 20 articles were included in the final list. Those who had surgery within 48 h had lower in-hospital and 30-day mortality rates than those who operated within 24 h. The American Society of Anesthesiologists (ASA) score is an important predictive factor for surgical delay, length of hospital stay (LOS), complications, and mortality. (4) Conclusions: Performing surgery in the first 48 h after admission is beneficial to patients after medical stabilization. Avoidance of delayed surgery will improve postoperative complications, LOS, and mortality.

## 1. Introduction

Hip fractures are currently recognized as a major public health problem, raising many issues in terms of both patients’ quality of life and the cost associated with caring for this type of fracture. Proximal femoral fractures (PFF) are more expensive than other types of osteoporotic fractures, with osteoporosis being blamed as one of the most important predisposing factors [1,2]. Regarding the cost of treatment, a report from the Australian and New Zealand Hip Fractures Registry states that in 2016, there were approximately 22,000 hip fracture patients in Australia alone, with a total cost of treatment estimated at $908 million [3]. For those patients suffering from a hip fracture, the painful and debilitating symptoms can have serious consequences by complicating pre-existing conditions, often leading to death [2]. In addition, the number of hip fractures has doubled in the last 30–40 years in many countries [1], with hip fractures occurring more frequently in Europe, Canada, and the United States, and it is expected for them to become more frequent globally as a result of demographic change [4]. It is well known that surgical treatment is the preferred therapeutic solution, as mortality for conservatively treated patients can be four times higher at one year and three times higher at two years after the fracture occurred, compared to surgically treated patients [5]. For the latter, the operating moment is intensely debated, which is the goal of a large number of studies addressing this topic. After studying the literature, we found that the articles present heterogeneous results without clear, punctual answers or solutions. Therefore, the aim of this review is to look for studies from the last few years and compare their results, trying to obtain a clear response regarding the optimum moment for surgery and its link to mortality.

## 2. Materials and Methods

### 2.1. Study Screening and Selection Criteria

The systematic search was performed according to the PICO guide (Patient/Problem, Intervention, Comparison group and Outcome) [6] using the Google Scholar platform. We also used the PRISMA guide (Preferred Reporting Items for Systematic Reviews and Meta-Analyses) [7]. The keywords used for this search were: “proximal femoral fracture surgical timing” and “hip fracture surgical timing”, among other synonyms. Two of the authors performed the searches in parallel. The inclusion criteria have been patients who suffered PFF (both intracapsular and extracapsular), over 18 years of age, hospitalized for the mentioned fracture, who underwent surgery, with the selected period being between 2015 and 2023. We did not include articles in other languages than English, studies involving a diaphyseal extension of the fracture, as well as those involving patients with associated fractures. Studies that did not present sufficient information on mortality, those that did not evaluate a possible correlation between mortality and the time to surgery, those that did not compare early versus delayed surgery, and those articles from which we did not have the possibility to extract data and group patients depending on the time to surgery (either 24 or 48 h) have been excluded. After this first thorough selection, the remaining items have been grouped according to the author’s definition of early and delayed operative time, namely 24 or 48 h. Finally, we studied the results regarding the time to surgery, mortality, the reasons for the delay, the length of hospitalization, the ASA grade (American Society of Anesthesiologists), and the complications.

The review has not been registered.

### 2.2. Quality Assessment

Two authors have used the Newcastle-Ottawa Scale (NOS) [8] to quantify the risk of bias in the selected studies. The authors checked the case definition, case representativeness, patient selection, and the definition of controls. Eventually, the score was subsequently used to determine the risk of bias.

### 2.3. Data Abstraction

The selected articles were evaluated by two of the authors to extract crucial data for the statistical analysis of this review. As stated above, studies that did not provide thorough information about the number of patients enrolled in each group (early/delayed) were excluded. The extracted information could characterize each cohort: number of patients, age, time to surgery, complications, mortality, and length of stay.

Regarding mortality, we obtained relevant data on how many patients died, separated for the early and delayed groups, and clustered the articles based on their endpoints: in-hospital mortality, 30-day mortality, and 1-year mortality. The odds ratio (RR) and confidence interval (CI) were calculated for every study using Epi.info, MedCalc, and Microsoft Excel. MedCalc has also been used for creating the forest plots.

For complications, patients were grouped into the following categories: urinary tract infection, deep venous thrombosis, pulmonary embolism, and wound infection. As has been done for mortality, we calculated the RR and CI. If the articles did not provide statistical information about the complications, their results were resumed narratively.

## 3. Results

### 3.1. Study Characteristics

**Figure 1:**
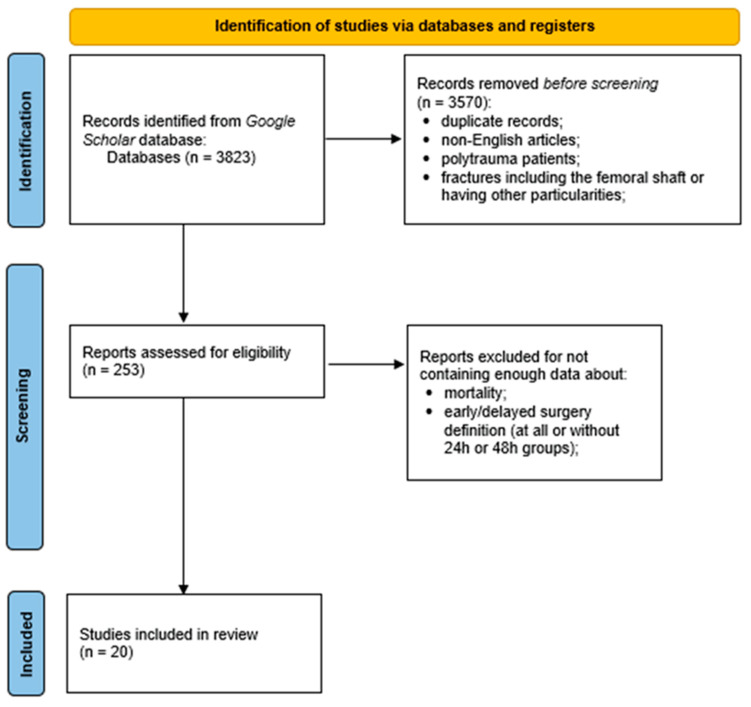
The PRISMA flow diagram for literature search in the systematic review.

After the inclusion and exclusion criteria were applied, 20 studies were included in the final list (Figure 1). Most of these were retrospective studies (15 studies). After calculating the risk of bias and analyzing the results of the revised studies, we found that 11 of them have a low risk of bias and 9 articles have a moderate risk (Table 1). Fourteen authors also presented results adjusted for different variables. The total number of patients analyzed in these studies was 272,402. Nine studies included only patients older than 65 years, three articles included patients older than 60 years, and two articles included patients older than 50 years; six authors did not mention the existence of age criteria in their studies.

### 3.2. Type of Fracture

The authors reported the fracture site in 14 studies (Table 1). Overall, intracapsular PFF was more common. On the other hand, in two articles, the authors did not mention the fracture location, but the type of surgery performed was noted. Maheshwari et al. reported that for 49.02%, closed/open reduction and internal fixation were performed for 45.41%—total/partial arthroplasty and other interventions performed for the remaining 5.55% [23]. Butler reported that hip arthroplasty was performed in 38.86% of cases [16]. Eleven other authors specified the surgical procedure [10,11,12,14,17,18,19,21,24,25,28].

### 3.3. Time to Surgery

In seven articles, early surgery was defined as surgery performed within 24 h after admission, and for the remaining articles, surgery performed within 48 h was defined as early (Table 1). Seven authors divided patients into several groups of time intervals [13,19,23,24,25,27,28]. Of all the patients included in the studies, 81,392 patients underwent surgery within the timeframe defined as early, and 190,760 patients were part of the delayed intervention group.

### 3.4. Reason for Delaying Surgery and the Preoperative Medical Condition of the Patient

The reason for delaying the intervention included, in general, medical optimization. Fifteen authors analyzed the patients according to the ASA score as well. This score is recognized as an important predictive factor, which influences the delay of surgery [9,12,13,15,16,22,23,27], the occurrence of postoperative complications [12,13,22], mortality [13,16,18,24], and the length of hospital stay [13,22]. It should be noted that Lizaur-Utrilla et al. showed that the ASA class influences mortality only by applying the univariate analysis [12], and in Maheshwari’s data, the ASA class does not influence mortality [23]. Two studies, Dong’s and Kristan’s, stated that the ASA score does not influence the surgical delay [11,21]. Butler et al. noted in their study that patients with preoperative episodes of clinical impairment waited 20.9 h longer for surgery (CI = 8.4–33.5; *p* = 0.001), and these episodes were correlated with a higher mortality at 30 days (14.9% compared to 2.3%; *p* = 0.001). At the same time, 90.2% of preoperatively decompensated patients had an ASA score ≥ 3 [16]. Shah et al. correlated the ASA score with the time of surgery, observing that most patients who underwent early surgery had an ASA class of 2 or 3, and most of those patients who underwent surgery after 48 h had an ASA class of 4 (*p* < 0.05) [9]. Lizaur-Utrilla et al. stated that an ASA score > 2 is a predictive factor for delaying surgery (*p* = 0.016 in multivariate analysis) [12]. Another author concluded that patients with an ASA score of 3–4 are nine times more likely to experience a delay in surgery than those with an ASA score of 1 [15]. In the article Maheshwari et al. wrote, 50.71% of patients operated on after 48 h had an ASA score ≥ 4 [23]. Kılınç et al., in their study of elderly patients (more than half over 80 years old—59.14%), reported that patients with an ASA score of 3 or 4 had a significantly greater mortality rate at one year than did those with an ASA score of 1 or 2 (78.30% vs. 58.40%; *p* < 0.001) [18].

### 3.5. Mortality

The information available regarding in-hospital mortality, 30-day mortality, and 1-year postoperative mortality was analyzed and grouped according to the 3 periods of follow-up, and eventually the RR and CI were calculated and compared. Nine authors reported data on in-hospital mortality [9,12,13,15,17,20,24,26,27], ten authors reported data on 30-day mortality [11,14,16,17,21,22,25,26,27,28], and seven authors reported information on 1-year mortality [10,12,15,16,18,21,23]. Mortality adjustment for at least one of gender, age, comorbidities (including ASA class), or operative time was performed in fourteen studies [9,10,12,13,14,15,18,19,21,22,23,25,27,28].

**Figure 2:**
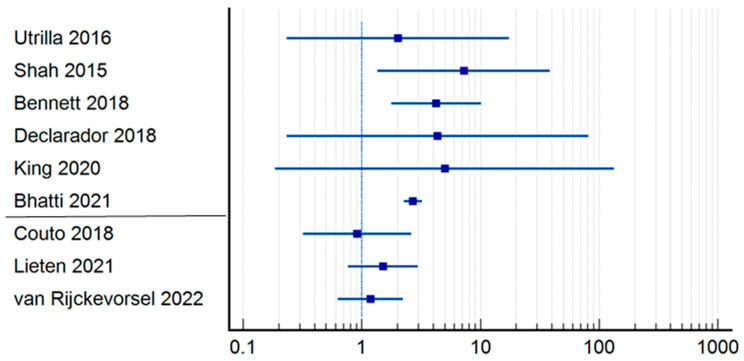
Odds ratio regarding in-hospital mortality [9,12,13,15,17,20,24,26,27].

In the group where in-hospital mortality was studied (Table 2) (Figure 2), we analyzed the relative risk in terms of delayed intervention—causality of death. Four studies had a statistically insignificant confidence interval [12,15,17,20,24]. However, the studies carried out by Bennett et al. (RR = 4.05; CI = 1.7398–9.4420; *p* = 0.0012) [13], Bhatti et al. (RR = 4.69; CI = 2.2616–3.2178; *p* < 0.0001) [20], and Shah et al. (RR = 6.63; CI = 1.3281–33.1433; *p* = 0.0211) [9] are worth mentioning, as they had statistically relevant results demonstrating the correlation between mortality and delayed surgery for more than 48 h, although their studies presented a moderate risk of systematic error. None of the three articles in this group that defined early surgery as occurring within 24 h demonstrated a statistically significant correlation between operative time and in-hospital mortality. After applying a numerical analysis to all the patients in the early surgery group, we found that 386 out of 24,097 patients who had the surgery performed within 48 h died, whereas 66 out of 1995 patients underwent surgery within 24 h (1.60% vs. 3.30%).

**Figure 3:**
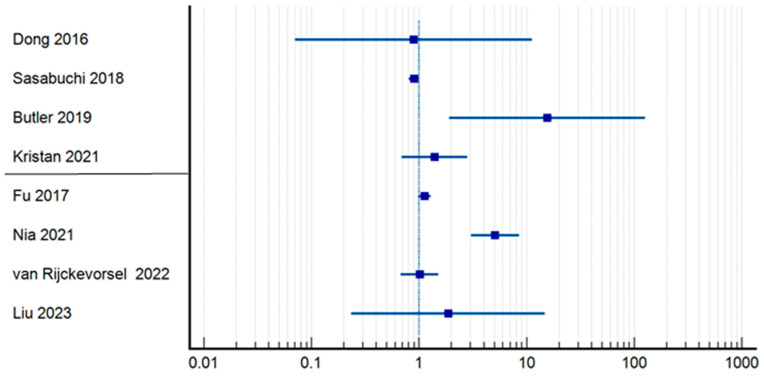
Odds ratio regrading 30-day mortality [11,14,16,21,22,25,27,28].

Regarding 30-day mortality (Table 3) (Figure 3), Butler et al. had the highest relative risk of mortality for patients who underwent delayed surgical treatment (RR = 14.31; CI = 1.8179–112.7351; *p* = 0.0115) [16], being followed by Nia et al. (RR = 5.09; CI = 3.0376–8.5555; *p* < 0.0001) [25], while Sasabuchi et al., the authors of a study that includes 208,936 patients, found that the time of surgery does not affect mortality at 30 days (RR = 0.90; CI = 0.8095–0.9989; *p* = 0.0476) [14]. The results of six other studies included in this group were not significantly different (*p* > 0.05) [11,17,21,22,27,28]. Similar to in-hospital mortality regarding the differences, the group undergoing surgery within 48 h experienced a lower mortality rate (0.99%) than did those who underwent surgery within 24 h (5.61%). Specifically, 475 out of 47,623 patients in the 48 h group died, whereas 448 out of 7972 patients in the 24 h group died.

**Figure 4:**
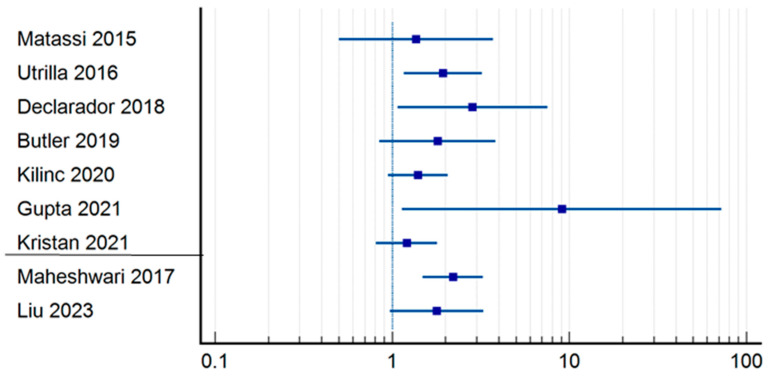
Odds ration regrading 1-year mortality [10,12,15,16,18,19,21,23,28].

One-year mortality (Table 4) (Figure 4) was influenced by the duration of surgery in the research papers performed by Gupta et al. (RR = 9.06; CI = 1.1364–72.3405; *p* = 0.0375) [19], Declarador et al. (RR = 2.67; CI = 1.0528–6.7721; *p* = 0.0386) [15], as was the case for Maheshwari et al. (RR = 1.87; CI = 1.3581–2.5878; *p* = 0.0001) [23]. It should be highlighted that the third article has the definition of early operative moment set at 24 h, and it is more statistically significant. The rest of the authors did not find a statistically significant correlation between the two variables analyzed [10,12,16,18,21,28]. Lizaur-Utrilla et al., on the other hand, observed that there was a difference in 1-year mortality between patients who underwent surgery within 4 days after admission and those who underwent surgery later (12.2% vs. 16.1%; *p* = 0.042) [12]. Analyzing the differences regarding the operating time in the early group, mortality was higher for those operated within 48 h, 185 of whom died out of a total of 1055, than for those operated under 24 h, as 54 patients out of 408 died (17.53% vs. 13.23%).

### 3.6. Complications

**Figure 5:**
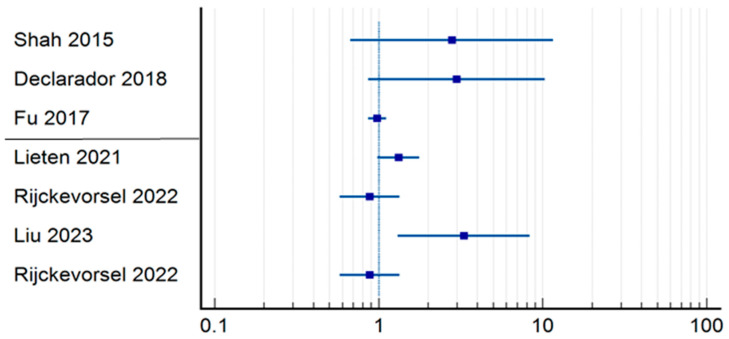
Odds ratio for developing urinary tract infectious for patients who had early surgery [9,15,22,26,27,28].

**Figure 6:**
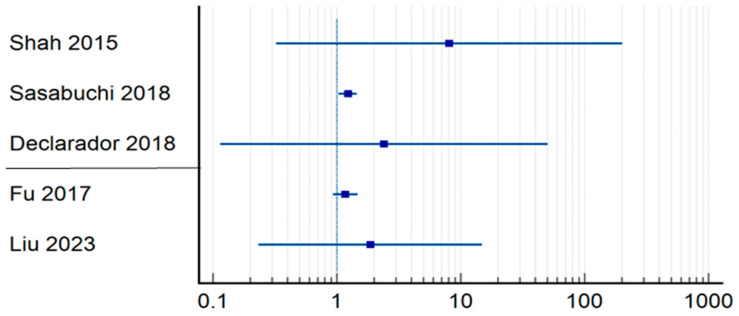
Odds ratio for developing an embolic event for patients who had early surgery [9,14,15,22,28].

**Figure 7:**
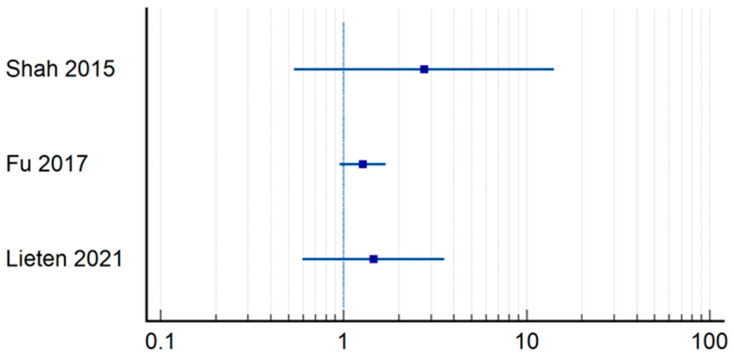
Odds ratio for developing wound infectious for patients who had early surgery [9,22,26].

Eleven authors collected data on postoperative complications [9,10,11,12,13,14,15,22,26,27,28], but only seven authors conducted a more concrete statistical analysis in an attempt to find a correlation with the timing of surgery [9,14,15,22,26,27,28] (Table 5). The most common complications that these authors tracked were urinary tract infections (Figure 5), thrombotic episodes (either pulmonary thromboembolism or deep vein thrombosis) (Figure 6), and wound infections (Figure 7). Only two authors in this group found a statistically significant link between the occurrence of complications and delayed surgery: Liu et al. for urinary tract infections (OR = 3.3; CI = 1.3084–8.3231; *p* = 0.0114) [28] and Sasabuchi et al. for thrombotic episodes (OR = 1.23; CI = 1.0397–1.4535; *p* = 0.0157). Furthermore, the latter also observed a higher presence of pressure ulcers in the delayed intervention group (1.6% vs. 1.0%, *p* < 0.001) [14]. Four authors found that delaying surgery favors pulmonary complications [14,22,26,28]. In the study performed by Fu et al., respiratory complications (including pneumonia, failure to extubate, or reintubation) also occur more frequently in patients undergoing delayed surgery (4.0% vs. 5.5%, *p* < 0.001; with propensity-adjusted OR = 0.78, CI = 0.67–0.90, *p* < 0.001) [22]. Moreover, this delay in intervention is a risk factor comparable to a CCI (Charlson Comorbidity Index) score of 6 for developing a postoperative respiratory complication. Another predictor of the occurrence of these pulmonary complications includes an ASA score ≥ 3 (OR = 2.14, CI = 1.69–2.70) [22].

Fu et al. showed that patients who underwent surgery within 24 h had a lower risk of developing any type of complication (15.6% vs. 17.3%; *p* = 0.003) [22]. Lizaur-Utrilla et al. found in a multivariate analysis a statistically significant correlation of postoperative complications with the ASA score only (*p* = 0.034) and in the univariate analysis the correlation was observed with the ASA score (*p* = 0.003) and with the CCI score (*p* = 0.041) [12]. Sasabuchi et al. correlated early intervention with a decreased risk of developing pressure ulcers (OR = 0.56, CI = 0.33–0.96; *p* = 0.035) [14], similar to the findings of van Rijckevorsel et al., who revealed that delaying surgery increases risk, especially when surgery is performed 48 h after admission (*p* = 0.002) [27].

### 3.7. Length of Stay

Less than half of the cited authors observed that the patients in the early group spent less time in the hospital than the patients in the group with delayed intervention, the information being statistically relevant [11,12,13,14,15,17,22,26,27]. Bennett et al. specify that the preoperative delay will obviously lead to a prolongation of postoperative hospitalization, which is why they also analyzed separately the days spent in postoperative hospital and observed an increase compared to patients who underwent an early surgery. The authors also reported that hospitalization has been directly proportional to the ASA class [13]. King’s et al. study was a comparison of two groups of patients on oral anticoagulant therapy (the groups were divided according to the time of surgery: early/delayed group) and a control group (patients without anticoagulant treatment operated within 48 h). The author found that postoperative hospitalization was prolonged in the delayed group, but not in the early group or the control group [17]. Lizaur-Utrilla et al. found that the total number of days of hospital stay was lower in patients who had early surgery (*p* = 0.001), but the mean postoperative hospital stay was not significantly different between the two groups (*p* = 0.242), which suggests that the length of stay was increased only in the context in which the operative moment was delayed [12]. Similarly, van Rijckevorsel et al. noted that there was no significant difference in postoperative length of stay between early and delayed surgery, although the overall LOS was longer when surgery was delayed (*p* < 0.001) [27]. In addition, Kılınç et al. observed that the length of hospital stay for patients who died within one year after surgery was significantly longer than that of survivors [18], without clearly mentioning a link between delayed surgery and longer hospitalization. In the study conducted by Fu et al., although delayed surgery is mentioned as a risk factor for increasing the length of stay, the influence of other variables is definitely stronger (medical conditions such as dialysis or a CCI score ≥ 7) [22]. In contrast to other studies that provide information on the length of hospital stay, Matassi et al. described a shorter hospital stay for patients who underwent surgery after 48 h (without statistical significance) [10].

## 4. Discussion

We noticed that over two-thirds of the operated patients included in these studies were part of the surgery group defined as delayed (70.09%). The timing of surgery was found to influence mortality rates in nine studies [9,13,15,16,19,20,23,25,28], with the statistical significance of this effect varying by study timeframe. One study found the effect to be significant within 30 days [16], while another observed significance over a 1-year period [28]. The rest of the authors did not find a statistically significant correlation between time of surgery and mortality, in compliance with our criteria. Depending on the follow-up period, only two in seven authors concluded that surgery within 24 h is beneficial for mortality [23,25], and six out of thirteen authors showed that surgery within 48 h is beneficial for mortality [9,13,15,16,19,20]. Typically, the benefit of surgery under 48 h should include the time frame between 24 and 48 h as well, although the findings of Maheshwari et al.’s results at one year postoperatively disagree with this presumption [23]. This is correlated with our observation that mortality at 1 year is not consistent with the results for in-hospital and 30-day mortality.

Following the comparison made on mortality between the patients belonging to the early group, it appears that those who had the surgery within 48 h had a lower in-hospital and 30-day mortality than those operated within 24 h. Thus, it is more appropriate for patients to be operated on within 48 h, compared to 24 h, considering that the priority should be stabilizing unstable patients and not rushing to surgery; overall, it is beneficial to patients to have the surgery performed under 48 h. At the same time, the inconsistent result on 1-year mortality compared to in-hospital mortality and 30-day mortality suggests that 1-year mortality cannot be a relevant outcome for these studies, as the time gap between surgery and death is too long to have them correlated, as other factors may intervene along the way.

Another endpoint followed was the occurrence of postoperative complications, and we observed that the delay of surgery is correlated with at least one postoperative event in seven studies, with results adjusted according to other factors [13,14,15,22,26,27,28].

The importance of the ASA score seems to be clear, as it is an important predictive factor for surgical delay [9,12,13,15,16,22,23,27], for the occurrence of complications [12,13,22], for mortality [13,16,18,21,24,25] and for the length of hospital stay [13,22]. The increased number of patients in the delayed group was also because many patients in this cohort had multiple comorbidities, which was reflected in the high ASA score and the need for optimized preoperative medical conditions. For example, Kristan et al. mentioned that patients with higher ASA require medical optimization, which has a positive effect on the outcome; he also found a correlation between a delay in surgery and patients with extracapsular PFF and high ASA [21]. Some of the authors correlated the delay of the surgery with an increase in the length of stay [11,12,13,14,15,17,22,26,27].

Declarador et al. mentioned that their result on in-hospital and 1-year mortality might be lower than in the literature, most likely due to the age of the patients and their better general condition, considering that patients with a poor health status were evaluated as unfit for anesthesia, and thus the selected treatment has been the conservative one [15]. Two authors considered that medical optimization should be prioritized for decompensated patients, as delaying the surgical time would not have negative results in these situations [11,13]. Lieten et al. propose the implementation of a complex protocol, managed together with the orthogeriatrician, adapted for patients with comorbidities that need stabilization, so that the intervention is performed within 24 h [26]. Maheshwari highlights that the impact of the operative moment may be different for healthy patients than for patients with comorbidities [23].

An issue observed during the analysis is that some researchers did not make a clear cohort distinction between patients with extracapsular or intracapsular fractures of the proximal femur, as both are included together in the study groups [10,11,12,14,15,17,18,20,26,28]. On the other hand, there are authors who did not declare the fracture site or the type of surgery. The two types of PFF may require different surgeries, meaning internal fixation or hip arthroplasty, which are dissimilar in terms of surgery—including the human resources, the surgical technique implied, and also the material resources [29,30,31]. Zeiter et al. wrote that the patients treated with arthroplasty may experience a delay in the surgery because of the absence of materials on stock or the requirement of closer supervision of the operator by the senior doctor [32]; Kristan et al. [21] and van Rijckevorsel et al. [27] similarly found that arthroplasty is a more complex procedure and is therefore predisposed to surgical delay. The authors of another study pointed out that intertrochanteric fractures are clearly more negatively influenced by the surgical delay than femoral neck fractures [33]. Therefore, the question arises as to whether the fracture site or the type of intervention may influence the outcome of the studies selected for this systematic review.

Among the authors reviewed in this paper, Sasabuchi et al. stated that early surgery was more common for patients with intertrochanteric fractures and those with internal fixation surgery [14]. Also, Couto et al. concluded that an arthroplasty procedure is a predictor of postoperative mortality [24]. In contrast, Nia et al. found that proximal femoral nailing and hemiarthroplasty were associated with higher 30-day and 180-day mortality rates compared to other surgeries (total hip arthroplasty, sliding hip screw, and double screw method), but did not offer an explanation for this result [25]. Chen et al. support this review`s idea and state that mortality and complications could be influenced by the type of fracture or intervention, but they did not have sufficient information to perform this analysis [34]. Moreover, Sheehan et al. showed that the type of intervention and the type of fracture are influencing the outcomes of in-hospital mortality [35].

In a study by Lefaivre et al., factors such as age, presence of comorbidities, type of fracture, and delayed surgery can significantly influence the length of hospital stay. They also mention that a delay of more than 24 h predisposes to the appearance of a minor medical complication, while a delay of more than 48 h increases the risk of a major medical complication and decubitus ulcers. However, the postponement of intervention did not significantly predict in-hospital mortality [36]. Another study found that for every 24 h of delay in surgery, the length of stay increases by 0.6 days; moreover, the waiting time for surgery is correlated with the presence of serious complications [37].

In a systematic review by Klestil et al., early surgery is associated with lower mortality as well as a lower complication rate. They found that studies that define early surgery within 48 h are associated with lower mortality [38]. In the same note, the results of another systematic review, conducted by Chen, show that mortality for those operated on both within 48 h and within 24 h is lower [34]. Those outcomes raise questions regarding the evolution of the group of patients who are operated on after 24 h, but under 48 h. Sheehan et al. concluded that operating time influences mortality, not alone but correlated to simultaneous factors that play at least the same important role [35]. In a comprehensive systematic review, Khan et al. highlighted that delaying the operating time may not affect mortality but may affect morbidity [39]. After comparing our statistical results, we tend to support this affirmation.

Furthermore, we found that the length of hospitalization plays a more important role in predicting mortality than time to surgery.

## 5. Limitations and Suggestions for Further Research

The results of these studies may be affected by the fact that the choice of patients` minimum age (as inclusion criteria) was at the discretion of each author, as well as the choice to include any type of hip fracture or any type of intervention (or the choice of not mentioning them at all). We also found that 1-year mortality results are not consistent with in-hospital or 30-day mortality, suggesting that this outcome might not be relevant or reliable. Due to the heterogeneity of the statistical results, we cannot state with certainty whether the time of surgery directly influences 30-day or 1-year mortality.

Therefore, in order to reduce the variability of the statistical analysis and to obtain clearer results, the future research methodology should group patients more closely: the results may be more relevant if patients were selected according to the ASA score (without grouping patients with ASA scores 1–2 and 3–4 simultaneously). Moreover, the patients should be clustered depending on the type of fracture or surgery, because the resources and treatment of extracapsular fractures differ from those for intracapsular fractures.

## 6. Conclusions

According to our analysis, performing surgery in the first 48 h after admission is generally beneficial to patients with no significant medical comorbidities. 

Morbidity is better correlated with time to surgery than mortality, and we consider it a better end point. Time to surgery influences the health state of patients with hip fractures (mostly frail patients) by prolonging LOS and increasing the risk of complications. The presence of comorbidities (quantified by the ASA score) may be a predictor of delayed surgery, postoperative complications, length of hospital stay, and mortality.

## Figures and Tables

**Table 1:** Study characteristics. CKD = chronic kidney disease, LOS = length of stay.

Author, Year of Publication, Country	Follow-Up	Sample Size	Extracapsular Fracture	Early Surgery Definition	Outcomes	Prolonged LOS for Delayed	Risk of Bias
Shah, 2015 Pakistan [9]	In-hospital	190	100%	48 h	In-hospital mortality/in-hospital complications	N/A	6
Matassi, 2015 Italy [10]	1 year	132	48.50%	48 h	1-year mortality/in-hospital complications/LOS/post-op functional ability	−	8
Dong, 2016 China [11]	30 days	28	15.62%	48 h	30-day mortality/30-day complications/LOS/post-op functional ability	+	4
Utrilla, 2016 Spain [12]	1 year	628	62.89%	48 h	In-hospital mortality/30-day mortality/3-month mortality/1-year mortality/complications	+	7
Bennett, 2018 USA [13]	In-hospital	841	N/A	48 h	In-hospital mortality/in-hospital complications	+	6
Sasabuchi, 2018 Japan [14]	30 days	208,936	48.80%	48 h	30-day mortality/30-day complications	+	7
Declarador, 2018 Singapore [15]	1 year	446	43.04%	48 h	In-hospital mortality/1-year mortality/In-hospital complications	+	7
Butler, 2019 Australia [16]	1 year	265	N/A	48 h	30-day mortality/1-year mortality/in-hospital complications	N/A	7
King, 2020 Australia [17]	90 days	84	55.95%	48 h	In-hospital mortality/30-day mortality/3-month mortality/in-hospital complications	+	7
Kilinc, 2020 Turkey [18]	1 year	443	51%	48 h	1-year mortality	N/A	6
Gupta, 2021 India [19]	1 year	87	79.31%	48 h	1-year mortality	N/A	8
Bhatti, 2021 USA [20]	In-hospital	28,031	N/A	48 h	In-hospital mortality/in-hospital complications	N/A	5
Kristan, 2021 Slovenia [21]	1 year	641	49.92%	48 h	30-day mortality/1-year mortality	N/A	8
Fu, 2017 USA [22]	30 days	26,051	N/A	24 h	30-day mortality/in-hospital complications	+	7
Maheshwari, 2017 USA [23]	1 year	720	N/A	24 h	1-year mortality	N/A	6
Couto, 2018 Portugal [24]	In-hospital	372	66.12%	24 h	In-hospital mortality	N/A	5
Nia, 2021 Austria [25]	180 days	1101	50.5%	24 h	30-day mortality/180-day mortality	N/A	8
Lieten, 2021 Belgium [26]	>2 years	840	N/A	24 h	In-hospital mortality/30-day mortality/in-hospital complications	+	6
van Rijckevorsel, 2022 The Netherlands [27]	30 days	1803	44%	24 h	In-hospital mortality/30-day mortality/in-hospital complications	+	8
Liu, 2023 China [28]	>2 years	763	43.9%	24 h	30-day mortality/3-month mortality/6 months mortality/in-hospital complications	−	5

**Table 2:** The effect of time to surgery on in-hospital mortality.

Study	Patient		In Hospital Mortality		Early Surgery (Hours)	Relative Risk	Confidence Interval
	Early	Delayed	Early n (%)	Delayed n (%)			
Shah, 2015 [9]	138	52	2 (1.44)	5 (9.61)	48	6.6346	1.3281–33.1433; *p* = 0.0211
Lizaur-Utrilla, 2016 [12]	180	448	1 (0.55)	5 (1.11)	48	1.9868	0.2337–16.8883; *p* = 0.5295
Bennett, 2018 [13]	575	266	8 (1.39)	15 (5.63)	48	4.0531	1.7398–9.4420; *p* = 0.0012
Declarador, 2018 [15]	144	302	0 (0)	4 (1.32)	48	4.3069	0.2334–79.4645; *p* = 0.3262
King, 2020 [17]	17	11	0 (0)	1 (9.09)	48	4.5	0.1995–101.5223; *p* = 0.3441
Bhatti, 2021 [20]	23,043	4419	375 (1.67)	194 (4.39)	48	2.6976	2.2616–3.2178; *p* < 0.0001
Couto, 2018 [24]	92	280	5 (5.43)	14 (5)	24	0.92	0.3406–2.4849; *p* = 0.8694
Lieten, 2021 [26]	517	288	19 (3.67)	16 (5.56)	24	1.5117	0.7655–2.9853; *p* = 0.2340
van Rijckevorsel, 2022 [27]	1386	362	42 (3.03)	13 (3.59)	24	1.1851	0.6294–2.2312; *p* = 0.5989
Total	26,092	6428	452 (1.73)	267 (4.15)			

**Table 3:** The effect of time to surgery on 30-day mortality.

Study	Patient		30-Day Mortality		Early Surgery (Hours)	Relative Risk	Confidence Interval
	Early	Delayed	Early n (%)	Delayed n (%)			
Dong, 2016 [11]	18	10	2 (11.11)	1 (10)	48	0.9	0.0927–8.7343; *p* = 0.9276
Sasabuchi, 2018 [14]	47,073	161,863	456 (0.96)	1410 (0.87)	48	0.8992	0.8095–0.9989; *p* = 0.0476
Butler, 2019 [16]	170	95	1 (0.58)	8 (8.42)	48	14.3158	1.8179–112.7351; *p* = 0.0115
King, 2020 [17]	17	11	0 (0)	1 (9.09)	48	4.5	0.1995–101.5223; *p* = 0.3441
Kristan, 2021 [21]	345	263	16 (4.63)	17 (6.46)	48	1.3938	0.6912–2.8104; *p* = 0.3534
Fu, 2017 [22]	5921	20,130	297 (5.01)	1129 (5.61)	24	1.1181	0.9872–1.2664; *p* = 0.0788
Nia, 2021 [25]	611	189	26 (4.25)	41 (21.69)	24	5.0979	3.0376–8.5555; *p* < 0.0001
van Rijckevorsel, 2022 [27]	1304	342	124 (9.5)	33 (9.64)	24	1.0147	0.6789–1.5167; *p* = 0.9432
Liu, 2023 [28]	136	657	1 (0.73)	9 (1.36)	24	1.863	0.2341–14.8270; *p* = 0.5566
Total	55,595	183,560	923 (1.66)	2608 (1.42)			

**Table 4:** The effect of time to surgery on 1-year mortality.

Study	Patient		1-Year Mortality		Early Surgery (Hours)	Relative Risk	Confidence Interval
	Early (n)	Delayed (n)	Early n (%)	Delayed n (%)			
Matassi, 2015 [10]	33	99	6 (18.18)	23 (23.23)	48	1.2778	0.5698–2.8652; *p* = 0.5519
Lizaur-Utrilla, 2016 [12]	180	448	26 (14.44)	61 (13.61)	48	0.9427	0.6161–1.4422; *p* = 0.7854
Declarador, 2018 [15]	144	302	5 (3.47)	28 (9.27)	48	2.6702	1.0528–6.7721; *p* = 0.0386
Butler, 2019 [16]	170	95	16 (9.41)	15 (15.78)	48	1.6776	0.8687–3.2399; *p* = 0.1234
Kılınç, 2020 [18]	212	231	69 (32.54)	93 (40.25)	48	1.237	0.9639–1.5874; *p* = 0.0948
Gupta, 2021 [19]	17	45	1 (5.88)	24 (53.3)	48	9.0667	1.1364–72.3405; *p* = 0.0375
Kristan, 2021 [21]	299	224	62 (20.73)	56 (25)	48	1.2056	0.8076–1.7999; *p* = 0.3603
Maheshwari, 2017 [23]	284	436	41 (14.43)	118 (27.06)	24	1.8747	1.3581–2.5878; *p* = 0.0001
Liu, 2023 [28]	124	561	13 (10.48)	105 (18.71)	24	1.7853	0.9718–3.2798; *p* = 0.0618
Total	1463	2441	239 (16.33)	523 (21.42)			

**Table 5:** The effect of time—surgery on the occurrence of complications.

Study (Early Definition, h)	Patient Group		Follow-Up	Patient Group		Odds Ratio	Confidence Interval
	Early (n)	Delayed (n)		Early (n)	Delayed (n)		
URINARY TRACT INFECTIONS							
Shah et al. [9]	138	52	In-hospital	4	4	2.7917	0.6717–11.6028; *p* = 0.1578
Declarador et al. [15]	144	302	In-hospital	3	18	2.9789	0.8630–10.2819; *p* = 0.0842
Fu et al. [22]	5921	20,130	30 days	338	1127	0.9796	0.8644–1.1102; *p* = 0.7470
Lieten et al. [26]	536	304	In-hospital	166	113	1.13187	0.9808–1.7730; *p* = 0.0670
Van Rijckevorsel [27]	1428	375	In-hospital	124	29	0.8814	0.5784–1.3432; *p* = 0.5570
Liu et al. [28]	137	666	In-hospital	5	74	3.3	1.3084–8.3231; *p* = 0.0114
DEEP VENOUS THROMBOSIS/PULMONARY EMBOLISM							
Shah et al. [9]	138	52	In-hospital	0	1	8.068	0.3234–201.2431; *p* = 0.2033
Sasabuchi et al. [14]	47,073	161,863	30 days	170	718	1.2293	1.0397–1.4535; *p* = 0.0157
Declarador et al. [15]	144	302	In-hospital	0	2	2.4043	0.1147–50.4087; *p* = 0.5720
Fu et al. [22]	5921	20,130	30 days	93	370	1.1734	0.9330–1.475; *p* = 0.1715
Liu et al. [28]	137	666	In-hospital	1	9	1.863	0.2341–14.8270; *p* = 0.5566
WOUND INFECTION							
Shah et al. [9]	138	52	In-hospital	3	3	2.7551	0.5380–14.1093; *p* = 0.2239
Fu et al. [22]	5921	20,130	30 days	58	250	1.2712	0.9539–1.6940; *p* = 0.1014
Lieten [26]	536	304	In-hospital	11	9	1.4561	0.5965–3.5544; *p* = 0.4092

## Data Availability

Not applicable.
